# Perda de altura radial em pacientes submetidos à fixação com placa bloqueada anatômica volar de rádio distal: Um estudo retrospectivo em uma instituição pública

**DOI:** 10.1055/s-0045-1814403

**Published:** 2025-12-30

**Authors:** Heitor Teixeira Alves Carvalho, Yves Pacheco Dias March e Souza, Felipe Pinheiro da Silva, Érica Maciel Heringer, João Carlos Ostermeir Silva Pereira, Saulo Fontes de Almeida

**Affiliations:** 1 Programa de Residência Médica, Instituto Nacional de Traumatologia e Ortopedia, Rio de Janeiro, RJ, Brasil; 2 Centro de Cirurgia da Mão, Instituto Nacional de Traumatologia e Ortopedia, Rio de Janeiro, RJ, Brasil

**Keywords:** fixação interna de fraturas, fraturas distais do rádio, placas ósseas, bone plates, fracture fixation, internal, radius fracture, distal

## Abstract

**Objetivo:**

Avaliar e quantificar a frequência e magnitude da perda tardia de parâmetros radiográficos de redução de fraturas de rádio distal em pacientes operados com placa volar bloqueada.

**Métodos:**

Este é um estudo retrospectivo e transversal que analisou radiograficamente 65 punhos operados entre 2020 e 2024 em uma instituição pública, avaliando a perda de altura radial no seguimento pós-operatório.

**Resultados:**

Os resultados mostraram uma perda mediana de 0,60 mm, com 13,8% dos pacientes apresentando perdas superiores a 2 mm e 75,4% perdas inferiores. Não encontramos indícios de que o sexo do paciente afete de forma estatisticamente significativa essa incidência após o teste de Wald

**Conclusão:**

Apesar da eficácia da placa volar bloqueada na manutenção da redução, a perda de altura radial ainda ocorre, especialmente em pacientes mais idosos e com fraturas mais complexas. O presente estudo corrobora a utilização dessa técnica de osteossíntese que mostra-se uma ferramenta fundamental na abordagem das complexas fraturas do terço distal do rádio, a fim de restaurar e manter a anatomia da região.

## Abstract (en)

**Objective:**

To evaluate and quantify the frequency and magnitude of late loss of radiographic parameters following volar locking plate fixation of distal radius fractures.

**Methods:**

The present is a retrospective, cross-sectional study that radiographically analyzed 65 wrists operated on between 2020 and 2024 at a public institution, evaluating postoperative loss of radial height.

**Results:**

The results showed a median loss of 0.60 mm, with 13.8% of patients experiencing losses greater than 2 mm, and 75.4% experiencing lower losses. No evidence was found that sex statistically significantly affects this incidence after the Wald test.

**Conclusion:**

Despite the effectiveness of the volar locking plate in maintaining reduction, loss of radial height still occurs, especially in older patients with more complex fractures. The present study corroborates the use of this osteosynthesis technique, a fundamental tool in the management of complex fractures of the distal third of the radius, to restore and maintain the anatomy of the region.

## Introdução

As fraturas da extremidade distal do rádio são as mais frequentes entre as fraturas dos membros superiores, correspondendo a 17% de todas as fraturas do esqueleto e 75% das fraturas do antebraço. 1 Apresentam uma distribuição bimodal, em jovens através de traumas de alta energia, e na população idosa com traumas de baixa energia. 2 Percebe-se, nos últimos anos, um aumento da prevalência das fraturas do rádio distal como resultado, principalmente, do envelhecimento populacional, representando o segundo tipo de fratura mais comum nessa faixa etária. 3

A análise específica das fraturas e o planejamento do tratamento requerem o conhecimento da anatomia normal da região. 4 A extremidade distal do rádio faz parte da articulação do punho junto à extremidade distal da ulna e aos ossos do carpo, havendo um equilíbrio anatômico e biomecânico entre as estruturas ósseas, músculo-tendinosas e ligamentares que permitem a funcionalidade da mão. 5

A extremidade distal do rádio possui uma anatomia complexa, com concavidades e inclinações em diferentes planos. Ela apresenta três facetas articulares côncavas, as fossas do escafoide e do semilunar e o entalhe sigmoide, onde articula-se com o escafoide, o semilunar e a cabeça da ulna, respectivamente. A fossa semilunar tem importância crítica na estabilidade das articulações rádio-cárpica e rádio-ulnar, envolvendo relações ósseas e ligamentares. 5 O aspecto dorsal da extremidade distal do rádio apresenta o tubérculo de Lister, uma proeminência óssea que pode variar de 4 a 10 mm e funciona como fulcro para o tendão extensor longo do polegar. 6

**Fig. 1:**
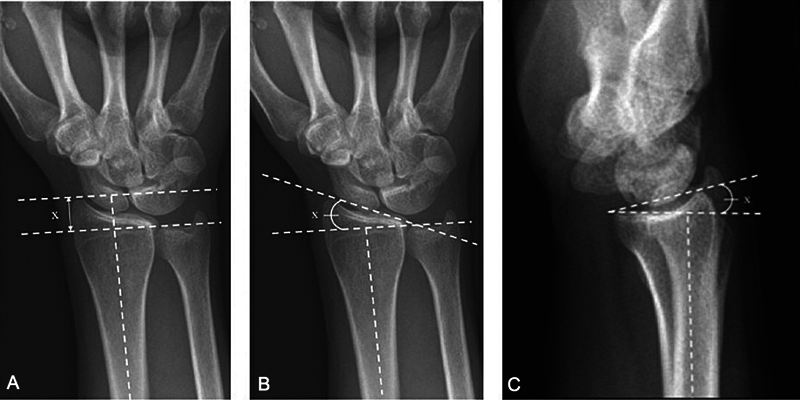
( **A** ) A altura radial média (X) é de 11 a 12 mm. ( **B** ) A inclinação radial média (X) é de 22 a 23 ^o^ . ( **C** ) O *tilt* volar (X) é de 11 à 22 ^o^ . (De Patel SP, Rozental TR. Distal radius fractures—biomechanics and classification. Hand Surgery Update VI. © 2016 American Society for Surgery of the Hand).

A extremidade distal do rádio possui inclinações e medidas que são avaliadas nas radiografias em incidências póstero-anterior (PA) neutra (inclinação radial, altura radial e variância ulnar) e de perfil (inclinação volar ou *tilt* volar) ( Fig. 1 ).

A inclinação radial é o ângulo entre uma linha tangenciando a superfície articular do rádio, desde o topo da estiloide radial ao canto ulnar do rádio e outra linha perpendicular ao eixo longitudinal da diáfise do rádio, com valor médio de 23 graus. 4

A altura radial, usada na avaliação do encurtamento do rádio após fratura, pode ser obtida pela incidência PA. Duas linhas são traçadas perpendicularmente ao eixo longo do rádio, uma na ponta do estiloide radial e a segunda na borda ulnar da superfície articular radial distal, seu valor normal é de cerca de 11 a 12 mm. 7

A variância ulnar é avaliada por uma linha tangente à superfície subcondral da articulação radiossemilunar e outra tangenciando a porção mais distal da superfície articular da ulna, com valor médio de 1 mm. 8

A inclinação volar ( *tilt* ) radial é o ângulo formado entre uma linha que tangencia os lábios volar e dorsal da fossa do semilunar e outra linha perpendicular ao eixo longitudinal do rádio, com valor médio de 11 graus. 4

A avaliação desses parâmetros de forma radiológica se provou um método confiável e reprodutível. 9

Esses parâmetros radiográficos são fundamentais para avaliar a correta restauração anatômica e funcional do terço distal do rádio após fraturas e suas respectivas reconstruções cirúrgicas. 10

As fraturas da extremidade distal do rádio possuem grande diversidade de padrões de apresentação e quando se requer o tratamento cirúrgico, embora várias técnicas já tenham sido descritas, a redução aberta e fixação interna com a placa volar bloqueada se tornou o método de escolha. 11 A técnica da placa volar bloqueada possibilitou a colocação de parafusos em posição subcondral, gerando sustentação para a superfície articular e diminuindo a perda de redução nas fraturas com cominuição dorsal. 12

A aplicação de placas bloqueadas na superfície volar do rádio permitiu a obtenção de fixação estável das fraturas intra e extra-articulares, possibilitando a mobilização precoce do punho e recuperação funcional articular mais rápida, tendo sido demonstrado superioridade nos resultados em relação às placas não bloqueadas, tanto volar quanto dorsal. 13 Apesar disso, apresentam taxas de complicações que variam de 3 a 36%, segundo a literatura. 14 Sendo a perda de redução a mais significativa entre as complicações possíveis. 15

Há um consenso entre cirurgiões de que os melhores resultados pós-cirúrgicos são obtidos através do restabelecimento dos parâmetros anatômicos radiográficos pré-operatórios. A perda de redução no pós-operatório, principalmente da altura radial, é uma complicação que pode levar à dor, instabilidade e perda de força. 16

Deste modo, este estudo tem como objetivo primariamente avaliar e quantificar a frequência e magnitude da perda tardia de parâmetros radiográficos de redução de fraturas de rádio distal em pacientes operados com placa volar bloqueada. Secundariamente, correlacionar os parâmetros radiográficos com a cronologia e os dados epidemiológicos dos pacientes.

## Materiais e Métodos

O presente estudo é uma análise retrospectiva e transversal, aprovada pelo Comitê de Ética em Pesquisa CAAE: 82364524.2.0000.5273, de pacientes com fratura de rádio distal submetidos à osteossíntese em nosso serviço entre os anos de 2020 e 2024. A placa bloqueada anatômica volar de rádio distal APTUS Wrist 2.5 (Medartis) foi utilizada como implante.

Os critérios de inclusão foram pacientes submetidos à cirurgia de rádio distal com APTUS Wrist 2.5, acompanhamento mínimo de 3 meses e paciente com idade superior a 18 anos.

Os critérios de exclusão foram pacientes com fraturas associadas no mesmo membro, uso de material de síntese adicional a placa bloqueada e pacientes sem registro radiográfico pós-operatório disponíveis no sistema de imagem digital.

Foram avaliados neste trabalho pacientes com tempo entre fratura e cirurgia de no máximo 4 semanas.

Por meio do filtro digital “FRATURA DA EXTREMIDADE DISTAL DO RADIO/ FRATURA DO ANTEBRAÇO”, foram selecionados os punhos operados em nosso serviço no período estabelecido. Após avaliação radiográfica e de prontuário, foram selecionados 114 punhos para o estudo.

As radiografias utilizadas são padronizadas pela instituição. Neste trabalho, utilizamos a incidência PA verdadeira com 1 metro de distância do *book* , 44 kilovolts (KV), e 3.2 de miliamperes por segundo (mA/s).

**Fig. 2:**
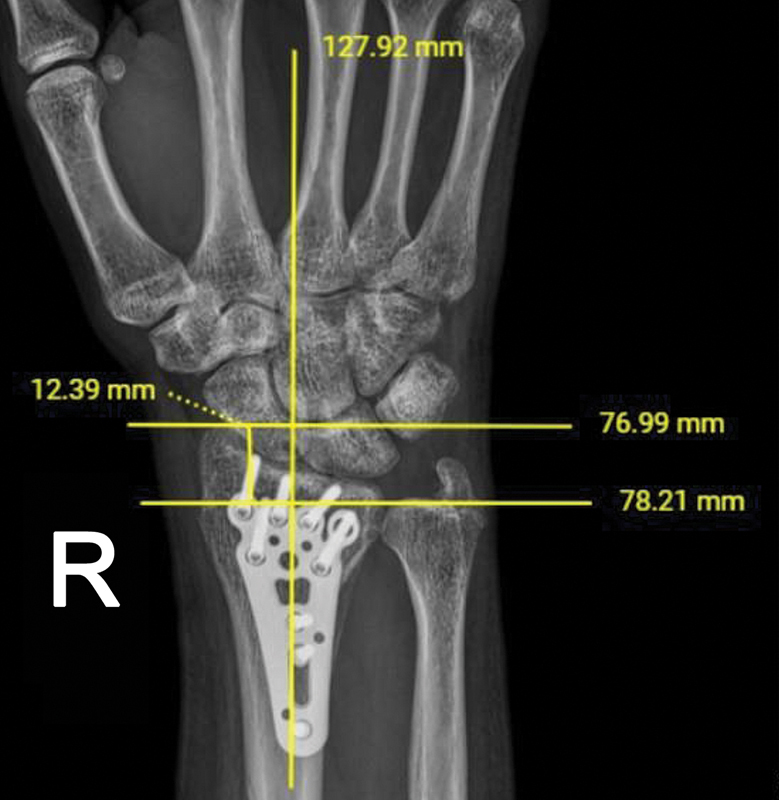
Altura radial no pós-operatório imediato.

Foi realizada a aferição da altura radial na primeira radiografia pós-operatória e nas radiografias pós-operatórias subsequentes através do sistema digital de imagens da instituição ( Figs. 2 3 ). Foram colhidos também dados como sexo, idade, lateralidade da fratura e data da cirurgia. Os dados obtidos foram planilhados e as aferições foram realizadas por quatro avaliadores.

**Fig. 3:**
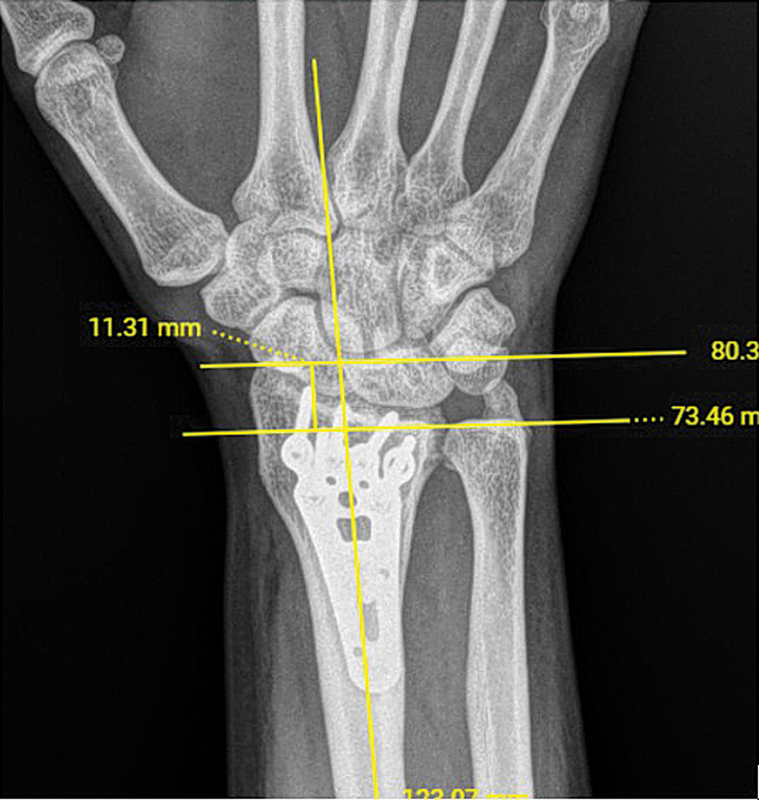
Alteração de altura radial em um período de 6 meses.

Os pacientes em pós-operatório de fratura do terço distal do rádio têm por protocolo de seguimento os retornos seriados em 3 semanas, 3 meses, 6 meses e 1 ano. Neste trabalho, recrutamos pacientes com pelo menos 2 radiografias no período de 3 meses.

Para padronização da avaliação utilizamos o sistema de classificação incongruência, deslocamento, energia, idade e lesões (IDEAL), cujas características são de análise de cinco variáveis. Para cada uma delas é conferida a pontuação de zero a 1, segundo ausência ou presença dos fatores, totalizando 5 pontos, sendo tipo I de 0 a 1 ponto, tipo II de 2 a 3 pontos e tipo III de 4 a5 pontos.

Para pontuar zero ou 1, foram utilizados os parâmetros de acordo com a Tabela 1 .

**Tabela 1:** Classificação IDEAL

	Característica	0 pontos	1 ponto
**I**	Idade	< 60 anos	> 60 anos
**D**	Desvio	Não	Desvio que necessita de redução
**E**	Energia 1	Baixa	Alta
**A**	Incongruência articular	Não	Incongruência ou *gap* > 2 mm
**L**	Lesões associadas 2	Ausentes	Presentes

**Notas** : 1. BAIXA: queda da própria altura/ALTA: outros.

2. Fraturas expostas/fratura dos ossos do carpo/instabilidade carpal/ ratura da ulna distal.

**Table:** 

Classificação	Escore	Descrição	Tratamento	Prognóstico
I	0–1 pontos	Estável	Conservador	Bom
II	2–3 pontos	Potencialmente instáveis	Fixação externa, pinagem percutânea e osteossíntese com placa	Intermediário
III	4–5 pontos	Complexa	Métodos associados, enxertia óssea	Ruim

## Análise estatística

Na análise, foi calculada a variação na altura radial em relação à primeira radiografia pós-operatória, expressa em milímetros. Os dados categóricos estão apresentados como ocorrência absoluta (percentual), enquanto os dados numéricos, que não apresentaram distribuição normal de acordo com o teste de Shapiro-Wilk (alpha de 0,05), estão apresentados como mediana (intervalo interquartílico [IIQ]). Foi calculada a incidência de perda na altura radial maior que 2 mm, e o efeito do sexo nessa incidência foi quantificado pela razão de chances (RC), obtida em regressão logística convencional, e avaliado pelo teste de Wald. As análises e figuras foram desenvolvidas em rotinas especialmente desenvolvidas em Python 3.10 (Python Software Foundation), com nível de significância de 0,05.

## Resultados

Foram analisados dados de 114 punhos, dos quais 65 tiveram acompanhamento mínimo de 3 meses e foram incluídos na análise. Os dados foram obtidos de 62 pacientes, sendo 3 casos de fratura bilateral.

**Fig. 4:**
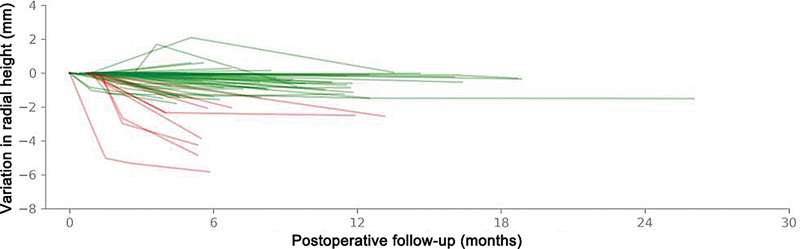
Variação na altura radial dos punhos acompanhados. Cada punho é representado por uma linha individual, sendo os punhos com perda de altura superior a 2 mm representados em vermelho, e os demais representados em verde.

Dentre os casos unilaterais, o lado acometido foi o esquerdo em 41 casos e o direito em 18. Os pacientes tinham 54 anos na mediana, com IIQ de 46 a 61, sendo 33 (53,2%) mulheres e 29 (46,8%) homens. O tempo de seguimento médio foi de 6 (IIQ 4–10) meses, variando de 3 a 18 meses ( Fig. 4 ).

**Fig. 5:**
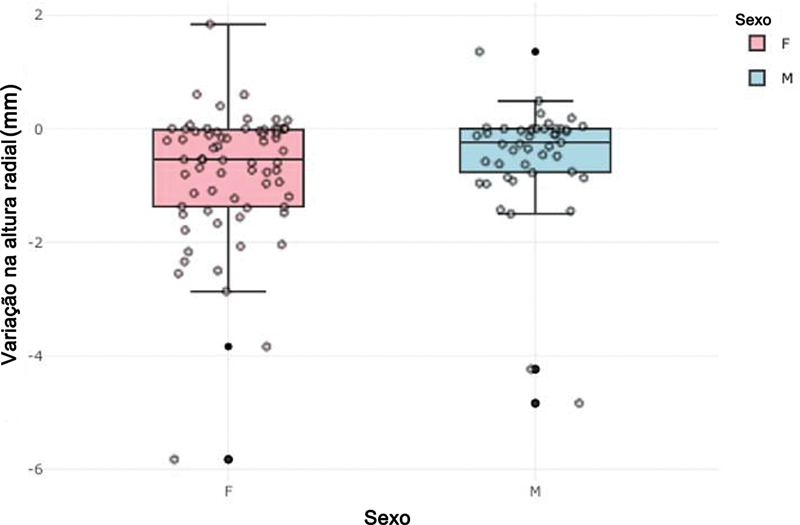
Variação pós-operatória da altura radial (em mm), por sexo.

Considerando a última medida obtida para cada punho, observamos uma perda de altura de 0,60 (0,14–1,43) mm, com um máximo de 5,83 mm ( Fig. 1 ). Não foi detectado qualquer nível de afundamento em 7 (10,8%) punhos, enquanto 49 (75,4%) apresentaram perdas detectáveis de até 2 mm e 9 (13,8%) apresentaram perdas maiores durante o período analisado ( Tabela 2 ). Foram observados 2 casos de perda maior que 2 mm entre homens e 7 entre mulheres, correspondendo a incidências de 6,7% e 20,0%, respectivamente. Não encontramos indícios de que o sexo afete de forma estatisticamente significativa essa incidência após o teste de Wald (RC = 3,5; *p* = 0,138) ( Fig. 5 ).

**Tabela 2:** Demonstrativo da amostra

	N	%
Sem perda de altura radia	7	10,8%
Perda de altura radial até 2 mm	49	75,4%
Perda de altura radial maior que 2 mm	9	13,8%
Total	65	100%

Utilizando a classificação IDEAL para avaliação dos pacientes e respeitando nossos critérios de inclusão e exclusão, foram avaliados pacientes apenas do tipo II. Isso deve-se ao fato de que no tipo I estão pacientes tratados de forma conservadora e no tipo III aqueles que necessitaram de métodos combinados de osteossíntese ou enxertia óssea. 17

Foram observados 2 casos de aumento de altura radial que retornaram ao parâmetro visualizado à primeira radiografia em medições subsequentes. Foram observados também outros 2 casos com apenas 2 radiografias de acompanhamento pós-operatório demonstrando aumento de altura radial maior que 1 mm (estes 4 casos estão descritos junto aos que não perderam altura radial). Este fenômeno pode ser explicado pela variabilidade da técnica de radiografia e foi também observado por outros autores em acompanhamentos radiográficos distintos. 18

## Discussão

A fratura da extremidade distal do rádio é uma das fraturas mais comuns em pacientes osteoporóticos e na população idosa. Com uma maior fragilidade, especialmente na porção metafisária do osso, a fixação destas fraturas é um desafio. Com o advento de placas bloqueadas anatômicas de rádio distal, promovendo estabilidade angular com os parafusos bloqueados, a manutenção da redução e possibilidade de reabilitação precoce é esperada pelos cirurgiões. Porém, estudos 19 demonstram que mesmo com o uso de placas bloqueadas, existe uma perda de parâmetros de redução pós-operatória.

Figl et al., 20 em 2009, avaliaram 58 pacientes com idade ≥ 75 anos por 13 meses, investigando perda secundária de redução após osteossíntese com placa volar bloqueada em pacientes com fratura de rádio distal, sem alteração no *tilt* volar. Cheng et al. 19 encontraram uma perda média de 1,3 ± 0,9 mm de altura radial na consulta de seguimento de 1 ano. O presente estudo aponta que 9% dos pacientes apresentaram uma perda média de altura radial de 1.3 mm, sem alterações na avaliação dos *tilts* radial e volar. Esses dados são condizentes com o que a literatura vem apresentando sobre o assunto.

Da mesma maneira, um estudo de Cheng et al. 19 avaliou 87 pacientes com fratura extra articular de rádio distal fixados com placa volar bloqueada, observando uma perda média de altura radial de 1.3 ± 0.9 mm. Segundo o autor, a idade avançada, variância ulnar positiva, as alterações na densitometria óssea e a menor distância entre o foco da fratura e a articulação radiocárpica foram associados à perda de altura radial. Neste estudo, 89,2% dos pacientes avaliados apresentaram algum grau de diminuição de altura radial, sendo que apenas 13,8% dos pacientes tiveram perda de altura radial maior que 2 mm.

A nossa amostra tinha uma média de idade de 54 anos, e considerando que a idade avançada é um fator de risco para perda da altura radial, observamos resultados semelhantes aos relatados na literatura quanto à perda média da altura radial. Uma justificativa para esse achado é a gravidade dos casos operados no nosso centro, uma vez que somos um centro de assistência quaternária no sistema de saúde. Com isso, observamos outros fatores de risco atuando indiretamente, tais como densitometria óssea e menor distância entre o foco da fratura e a articulação radiocárpica e o grau de fragmentação. 15 21

Thompson 22 descreveu em sua revisão sistemática a presença de outras complicações possíveis, bem como: síndrome do túnel do carpo (2–3%), ruptura tendinosa (1–2%) e necessidade de remoção do material de síntese em (6%). Além disso, o estudo de Xavier et al. 23 apontou que a perda da altura radial resultou em um déficit de força de preensão associado a perda de arco de movimento.

De acordo com a definição da classificação IDEAL, fraturas avaliadas como tipo II apresentam desvio e são consideradas potencialmente instáveis. Possuem alto risco de perda da redução e de evolução para consolidação viciosa, seja pela baixa qualidade óssea em pacientes idosos, pelo trauma de alta energia em jovens, pela presença de incongruência articular ou ainda por lesões associadas em qualquer faixa etária. 17 Nesse contexto, conseguimos observar que nesse grupo, a perda da altura radial no seguimento pós-operatório acontece, mas, em média, é menor que 2 mm.

Farhan et al. 24 demonstraram que pacientes com casos mais complexos, tratados com associação de métodos de osteossíntese, apresentaram uma média de altura radial de 8,5 mm. Isso representa uma perda maior que 2 mm. Sendo assim, concluimos que em casos definidos como tipo III pelo sistema de classificação IDEAL há uma maior alteração da altura radial.

O presente estudo, com um bom número de paciente avaliados e com a utilização de um mesmo modelo de placa bloqueada, outorga confiabilidade aos resultados obtidos. Indo ao encontro à literatura atual sobre o assunto, corroborando e incentivando ao uso de placa bloqueada para fraturas de rádio distal a fim de buscar restaurar e manter os parâmetros anatômicos da região.

No entanto, é fundamental trazer luz aos pontos negativos. A perda de seguimento de 42.9% dos pacientes da amostra inicial mostra-se uma dificuldade presente em serviços públicos, além do relevante número de pacientes que foram operados com um tempo não ideal de fratura, superior a 4 semanas, excluídos da amostra. Ademais, há como perfil das fraturas e dos pacientes operados nessa instituição (um serviço quaternário) um possível viés de seleção, e também não foi realizada uma avaliação/subdivisão dos casos quanto aos seus parâmetros pré-operatórios, podendo ser feita essa análise em artigos futuros.

Nosso artigo possui alguns pontos positivos. O primeiro, um bom número de pacientes avaliados, conferindo maior confiabilidade aos resultados obtidos. Segundo a utilização de um mesmo modelo de placa bloqueada, interferindo o mínimo possível na técnica cirúrgica e posterior análise. Por fim, uma homogeneidade da amostra, dada a avaliação de pacientes classificados apenas como tipo II da classificação IDEAL, diminuindo vieses que poderiam confundir os resultados. No entanto, é fundamental trazer luz aos pontos negativos observados, como: a variação da gravidade das fraturas analisadas e tempo curto de seguimento. Além disso, não houve uma avaliação funcional dos pacientes correlacionada com os parâmetros radiográficos, uma vez que não era o objetivo principal do trabalho, sendo uma possibilidade para estudos futuros. Assim, torna-se fundamental a continuidade do acompanhamento para consolidação dos resultados e análises de trabalhos futuros.

## Conclusão

Apesar de placas bloqueadas anatômicas de rádio distal oferecerem estabilidade angular aos parafusos bloqueados, é esperada uma perda de altura radial no seguimento pós-operatório. Porém, na maior parte dos pacientes é esperado que essa perda seja menor que 2 mm. O presente estudo corrobora a utilização dessa técnica de osteossíntese que mostra-se uma ferramenta fundamental na abordagem das fraturas do terço distal do rádio a fim de restaurar e manter a anatomia da região. Por fim, mais estudos são fundamentais para a avaliação correlacional entre funcionalidade e parâmetros radiográficos.
